# A Study on Myogenesis by Regulation of Reactive Oxygen Species and Cytotoxic Activity by Selenium Nanoparticles

**DOI:** 10.3390/antiox10111727

**Published:** 2021-10-29

**Authors:** Sang-Cheol Lee, Na-Hyun Lee, Kapil D. Patel, Soo-Kyung Jun, Jeong-Hui Park, Jonathan Campbell Knowles, Hae-Won Kim, Hae-Hyoung Lee, Jung-Hwan Lee

**Affiliations:** 1 Institute of Tissue Regeneration Engineering (ITREN), Dankook University, 119 Dandae-ro, Cheonan 31116, Chungcheongnam-do, Korea; 72210656@dankook.ac.kr (S.-C.L.); nhlee0609@dankook.ac.kr (N.-H.L.); dynamic2020@korea.ac.kr (K.D.P.); shurins@naver.com (J.-H.P.); j.knowles@ucl.ac.uk (J.C.K.); kimhw@dku.edu (H.-W.K.); 2 Department of Biomaterials Science, College of Dentistry, Dankook University, 119 Dandae-ro, Cheonan 31116, Chungcheongnam-do, Korea; 3 Department of Nanobiomedical Science & BK21 PLUS NBM Global Research Center for Regenerative Medicine, Dankook University, 119 Dandae-ro, Cheonan 31116, Chungcheongnam-do, Korea; 4 Department of Materials Science and Engineering, Korea University, Seoul 02841, Korea; 5 Department of Dental Hygiene, Hanseo University, Seosan 31962, Korea; iris979@hanseo.ac.kr; 6 UCL Eastman-Korea Dental Medicine Innovation Centre, Dankook University, 119 Dandae-ro, Cheonan 31116, Chungcheongnam-do, Korea; 7 Division of Biomaterials and Tissue Engineering, Eastman Dental Institute, University College London, London WC1E 6HH, UK; 8 Cell & Matter Institute, Dankook University, Cheonan 31116, Chungcheongnam-do, Korea; 9 Department of Regenerative Dental Medicine, College of Dentistry, Dankook University, Cheonan 31116, Chungcheongnam-do, Korea; 10 Mechanobiology Dental Medicine Research Center, Cheonan 31116, Chungcheongnam-do, Korea

**Keywords:** C2C12, skeletal muscle differentiation, selenium nanoparticle, ROS, antioxidant

## Abstract

Reactive oxygen species (ROS) are continuously produced by skeletal muscle during contractile activity and even at rest. However, the ROS generated from excessive exercise or traumatic damage may produce more ROS than can be neutralized by an antioxidant capacity, which can be harmful to muscle function. In particular, selenium is a known antioxidant that regulates physiological functions such as cell differentiation and anti-inflammatory function. In this study, we developed nano-sized antioxidative biomaterials using selenium to investigate the protective and differentiation effects against C2C12 myoblasts in an H_2_O_2_-induced oxidative stress environment. The selenium nanoparticles (SeNPs) were produced with a size of 35.6 ± 4.3 nm and showed antioxidant effects according to the 3,3′,5,5′-tetramethylbenzidine assay. Then, SeNPs were treated to C2C12 cells with or without H_2_O_2_. Our results showed that SeNPs reduced C2C12 apoptosis and intracellular ROS levels. Additionally, SeNPs effectively up-regulated in the presence of H_2_O_2_, *MyoD*, *MyoG*, *α-actinin*, and myosin heavy chain, which are well known to increase during myoblast differentiation as assayed by qRT-PCR, immunocytochemistry-staining, western blotting. These results demonstrate that SeNPs can accelerate differentiation with its protective effects from the ROS environment and can be applied to the treatment of skeletal muscle in a cellular redox environment.

## 1. Introduction

Skeletal muscle is a highly contractile tissue that makes up a large portion of the total human body mass (40–50%). Skeletal muscle possesses excellent regenerative capacity upon damage to adapt to various physiological conditions due to their essential role in posture and movement [1,2,3,4]. Mainly, the resident skeletal muscle stem cells, called satellite cells, control skeletal muscle tissue regeneration. Satellite cells differentiate into myogenic cells and fuse to each other to form a bundle of multinucleated myoblasts, which are mature myofibers [5,6].

During physical activity, skeletal muscle constantly generates reactive oxygen species (ROS) due to their metabolic activity. ROS modulates cellular proliferation, migration, differentiation, and muscle contractions at the physiological level [7,8]. However, prolonged exposure to excessive ROS possibly exceeds the muscle’s antioxidant capacity and may result in damage and injury, including irreversible fibrosis and scarring, which can eventually cause loss of muscle function [9]. Unfortunately, no surgical reconstructions or therapeutic interventions allow for complete functional restoration [10]. Thus, the development of therapeutic strategies that treat skeletal injuries is required.

Recently, there have been many reports on the development of biomaterials for tissue regeneration [11,12,13,14,15,16]. Importantly, biomaterials for tissue-engineered skeletal muscle provide a biophysical microenvironment to help myoblast differentiation and tissue formation [17,18,19]. In particular, nanoparticles with diameters of 1–100 nm can modulate cell fate by directly delivering themselves to intracellular sites, such as to the cytosol and nucleus [20,21,22]. Recently, metal (gold, silver, selenium, strontium, copper, and ceria)-incorporated nanoparticles showed outstanding biocompatible and antioxidant effects in various areas of tissue regeneration [20,23,24,25,26,27].

Selenium is one of the essential trace elements that regulate various physiological functions, including anti-inflammatory effects and immune functions [28,29,30,31], and is also well known to play an essential role in regulating ROS levels [32,33,34,35]. Selenium has been reported to protect fibroblasts from oxidative stress [36] and apoptosis [37,38,39,40,41]. Nanoscale selenium is a highly effective molecular compound with higher antioxidant activity and lower toxicity than regular selenium [42]. Therefore, in this study, we focused on the antioxidant effects of SeNPs on skeletal muscle cell differentiation in a ROS exposure environment.

## 2. Materials and Methods

### 2.1. Materials

Sodium selenite (Na_2_SeO_3_; Sigma–Aldrich, St. Louis, MO, USA), L-ascorbic acid (C_6_H_8_O_6_, Sigma, USA), D-(+)–glucose (C_6_H_12_O_6_, Sigma, USA), sodium hydroxide beads (NaOH, Daejung, Seoul, Korea), and 1N- hydrochloric acid (HCL, Daejung, Korea) were used. All chemicals were reagent-grade and used without further purification.

### 2.2. Synthesis of Selenium Nanoparticles (SeNPs)

SeNPs were synthesized by the reduction method, as previously reported by our group [26]. Briefly, an aqueous solution of sodium selenite (100 mM) was mixed with an aqueous solution of ascorbic acid (100 mM) using magnetic stirring, and the pH was maintained at 7.1 using NaOH (0.1 M) or HCl (0.1 M). The resulting mixture solution color changed from transparent to red and the reaction was carried out for 6 h. Finally, SeNPs were collected by centrifugation at 15,000 rpm for 30 min and washed with deionized water three times. The final solution was lyophilized to collect the SeNPs.

### 2.3. Characterizations of SeNPs

The morphology and size, crystalline phase, chemical functional groups, surface charge, and optical properties of SeNPs were characterized by transmission electron microscopy (TEM; JEOL-7100, JEOL, Tokyo, Japan), X-ray diffraction (XRD; Rigaku, Tokyo, Japan), Fourier transform infrared spectroscopy (FTIR; Varian 640-IR, CA, USA), zeta potentials (Zetasizer Nano; Malvern Instrument, Worcestershire, UK), and UV-visible analysis (UV-vis, Varian Cary 100, Agilent Technologies, Paolo Alto, CA, USA), respectively.

### 2.4. Peroxidase Activity of SeNPs

We investigated the oxidase-like activity of SeNPs, as previously reported by our group [26]. Briefly, the catalytic activity (i.e., oxidation of the peroxidase substrate) of SeNPs was observed in 3,3′,5,5′-tetramethylbenzidine (TMB) in the presence of hydrogen peroxide (H_2_O_2,_ Sigma–Aldrich, USA). Two hundred micrograms of SeNPs were dispersed in 200 mL of 200 µg/mL TMB solution mixed with H_2_O_2_ and placed at room temperature for different times (0 h, 1 h, and 5 h) in the dark, after which optical images were taken. After the predefined reaction time, the supernatant for each sample was collected via centrifugation and the UV-visible absorption (400–800 nm) range was measured to verify the oxidase-like activity of the SeNPs.

### 2.5. Cell Culture

C2C12 mouse myoblast cells were maintained at 37 °C in a 5% CO_2_ atmosphere in Dulbecco’s modified Eagle’s medium (DMEM, Welgene, Dalseogu, Daegu, Korea). Unless otherwise specified, the medium contained 10% heat-inactivated fetal bovine serum (FBS, Corning, Woodland, CA, USA), 100 U/mL of penicillin, and 100 μg/mL of streptomycin (PS, Gibco, Grand Island, NY, USA).

Cell medium was changed every two days. When C2C12 cells reached 70% confluence, they were detached by treatment with 0.25% trypsin EDTA (Gibco, Grand Island, NY, USA) and replated for experiments. The cells used in all experiments were between 8 and 10 passages. For myogenic differentiation, cells were seeded at a density of 4 × 10^4^ in 24-well plates. After 24 h of seeding, cells were treated with media containing SeNPs and then H_2_O_2_ was added to cells after 30 min. Cell culture media were replaced 24 h later with myogenic differentiation media consisting of DMEM with 1% PS and 2% horse serum (Gibco, Grand Island, NY, USA).

### 2.6. Cell Viability

In vitro cell viability on the SeNPs or H_2_O_2_ was studied. C2C12 cells were seeded into a 96-well plate at 5 × 10^3^ cells per well. After 24 h, either SeNPs (0–320 μg/mL) or H_2_O_2_ (0–1000 μM) was treated for 1 day. Then, the cell viability assay was performed using a cell counting kit-8 (CCK-8, Dojindo, Kumamoto, Japan). Briefly, the culture medium containing 10% CCK-8 solution was replaced in each well of the plate, followed by incubation for 2 h at 37 °C. After that, the absorbance of each well was read at 450 nm using a microplate reader (Thermo Fisher Varioskan LUX, Waltham, MA, USA). Cell survival was also examined by live/dead-staining (0.5 μM of calcein AM and 2 μM of ethidium homodimer-1 solutions, Thermo Fisher, Waltham, MA, USA) and images were taken using an optical microscope (IX71, Olympus, Tokyo, Japan). After staining, the rate of live cells (%) was quantified using ImageJ software (Bethesda, Maryland, USA).

To examine the cellular cytotoxicity under both SeNPs and H_2_O_2_, C2C12 cells were plated in a 96-well plate at a seeding density of 5 × 10^3^ cells. Then, SeNPs (0–80 μg/mL) were treated to cells with or without H_2_O_2_ (100 or 200 μM) for 1 day. The cell viability assay and live/dead-staining were performed as we described above.

### 2.7. ROS-Staining

High oxidative stress conditions were enabled by pretreatment with 500 μM of H_2_O_2_ for 4 h as previously reported by our group [26]. ROS levels were analyzed using the Image-iT LIVE Green Reactive Oxygen Species Detection Kit (Invitrogen, Carlsbad, CA, USA). The cells were gently washed twice with HBSS/Ca/Mg (Welgene, Dalseogu, Daegu, Korea) and labeled with 25 μM of carboxy-H2DCFDA to cover the adherent cells for 30 min at 37 °C. The labeled cells were gently washed three times and observed using a microscope.

### 2.8. qRT–PCR

C2C12 cells were cultured with or without SeNPs and H_2_O_2_ for 5 days. Total RNA was harvested using an RNA preparation kit (Geneall, Songpa-gu, Seoul, Korea) according to the manufacturer’s instructions and the RNA concentration was analyzed using a Nanodrop (Thermo Fisher, Waltham, MA, USA). First-strand cDNA was developed using an RNA reverse transcription (RT) kit (Bioneer, Daeduk-gu, Daejeon, Korea) according to the manufacturer’s instructions. PCR was performed using target gene expression levels normalized to glyceraldehyde 3-phosphate dehydrogenase (*Gapdh*) levels. The delta cycle threshold (Ct) method was used to calculate relative levels of expression. The primer sequences were as follows: Myogenic determinant (*MyoD)* forward 5′-GGA GTG GCA GAA AGT TAA G-3′, reverse 5′-ACG GGT CAT CAT AGA AGT C-3′; Myogenin *(MyoG)* forward 5′-GGA TAT GTC TGT TGC CTT C-3′, reverse 5′-TGG GTG TTA GCC TTA TGT-3′; *Alpha actinin (α-Actinin)* forward 5′-GGA CTA CAC TGC CTT CTC-3′, reverse 5′-CAG CCT ATA CTT CAG CCT TT-3′; and *Gapdh* forward 5′-GGT TGT CTC CTG CGA CTT CA-3′, reverse 5′-TAG GGC CTC TCT TGC TCA GT-3′.

### 2.9. Myotube Formation and Immunocytochemistry

C2C12 cells were seeded into 24-well plates at a density of 4 × 10^4^ cells/well; after 24 h, cells were treated with SeNPs (5–20 μg/mL). After 30 min of treatment with SeNPs, H_2_O_2_ was added to maintain high oxidative stress conditions and myogenic differentiation medium was added for 5 days to induce myotube formation. The differentiation medium was changed every other day. On the 5 days of differentiation, the cells were washed with PBS (Tech and Innovation, Chuncheon, Korea), fixed with 4% paraformaldehyde (PFA, Tech-innovation, Gangwon-do, Korea) for 30 min, and permeabilized with 0.2% Triton X-100 (Sigma–Aldrich, USA) for 10 min. After permeabilization, the cells were blocked with 1% BSA solution for 30 min, incubated with Myosin heavy chain antibody (MHC: 1:400, Santa Cruz Biotechnology, Inc., Dallas, TX, USA) at 4 °C overnight, and incubated with secondary antibody (FITC, anti-mouse 1:200; Abcam Inc., Cambridge, UK) at room temperature for 2 h. The multinucleate myotubes were observed using a fluorescence microscope (CELENA, Logos Biosystems, Anyang, Korea).

### 2.10. Western Blot

C2C12 cells were cultured in differentiation media and treated with SeNPs (5 or 10 μg) in the presence of 50 μM of H_2_O_2_ for 5 days as we described above. Then, it was dissolved in RIPA lysis buffer (ELPIS Biotech, Daejeon, Korea) containing protease inhibitor (Halt™ Protease and Phosphatase Inhibitor Cocktail (100×), Thermo Fisher Scientific, Waltham, MA, USA). The total protein concentration was quantified using a Pierce BCA protein assay kit (Thermo Fisher Scientific, Waltham, MA, USA). An equal amount of total proteins per lane was electrophoresed in polyacrylamide gels and transferred onto polyvinylidene difluoride (PVDF, Bio-Rad Laboratories, Hercules, CA, USA) membranes. The membranes were blocked with 5% bovine serum albumin (BSA, BD Biosciences, Sparks, MD, USA) in TBS-T solution (Tris-buffered saline-0.1% Tween, LPS Solution, Daejeon, Korea) for 1 h and then incubated with primary antibodies in 5% (*w*/*v*) BSA in TBS-T solution at 4 °C overnight against MYOD (myoblast determination protein 1:1000, Cell Signaling Technology, Danvers, MA, USA), MYOG (1:1000, Millipore, Billerica, MA, USA), BCL-2 (1:1000, Cell Signaling Technology), BAX (1:1000, Abcam, Cambridge, United Kingdom), or β-ACTIN (Cell Signaling Technology). After washing with TBS-T, the membranes were further incubated with secondary antibodies at room temperature for 1 h. The signals were detected using a western blot imaging system (ibright FL1500, Invitrogen, Carlsbad, CA, USA) and the band intensity was normalized to β-ACTIN as well as quantified by ImageJ software (National Institutes of Health, Bethesda, MD, USA).

### 2.11. Statistical Analysis

Statistical significance between groups was evaluated by one-way ANOVA followed by Dunnett’s multiple comparisons tests or by two-way ANOVA, followed by Dunnett’s multiple comparisons tests. GraphPad Prism 8 software (San Diego, CA, USA) was used.

## 3. Results and Discussion

### 3.1. Characterization of SeNPs

**Figure 1:**
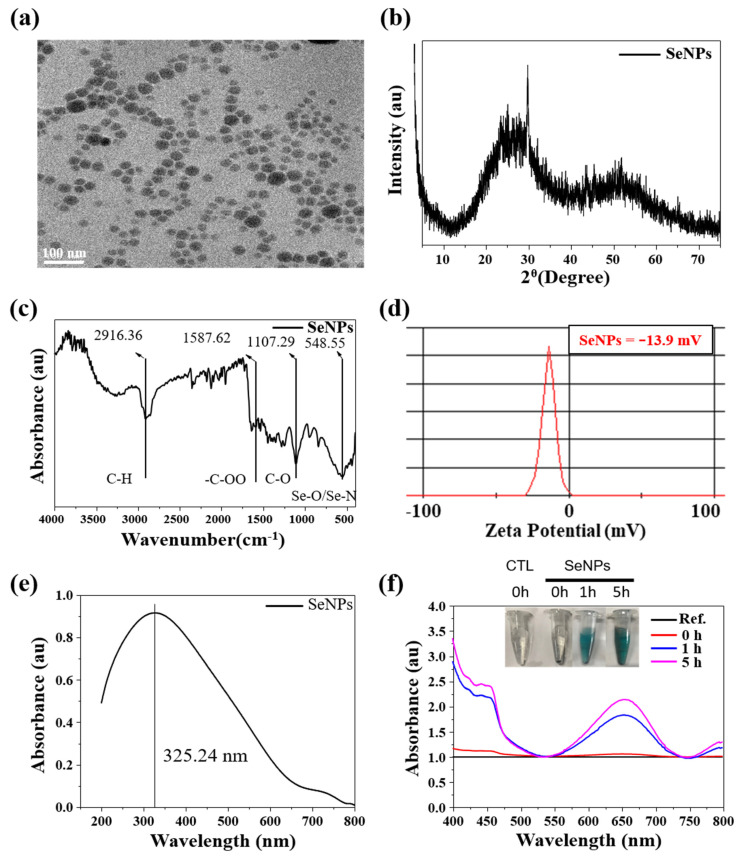
Characterizations of SeNPs. (**a**) TEM image of SeNPs, (**b**) XRD patterns, (**c**) FT-IR spectra of SeNPs, (**d**) ζ-potentials of SeNPs, and (**e**) UV-visible spectra of SeNPs in DW. (**f**) Optical image of the SeNPs reaction with TMB at 0 h, 1 h, and 5 h; the corresponding change in color (SeNPs concentration in 200 µg/mL and TMB concentration in 200 µg/mL) at pH 4.0 in DW; and UV-visible absorption spectra of TMB (before reaction as a reference) and after reaction for 0 h, 1 h, and 5 h with and without SeNPs.

Characteristics0 of SeNPs were investigated to check as-designed size, shape, and chemical composition. The HR-TEM image of SeNPs is shown in Figure 1a. The SeNPs were spherical, with a size of 35.6 ± 4.3 nm. Next, we investigated the crystal phage structure of the SeNPs. The XRD patterns of SeNPs are shown in Figure 1b and they exhibited two broad peaks at 2θ = 20–30° and 45–55°, demonstrating a combination of crystalline and amorphous phases of the nanoparticles [43]. The FTIR spectra of the SeNPs are presented in Figure 1c. The sharp and intense peak at 2916.36 cm^−1^ corresponds to the –CH group, the peak at 1587.62 cm^−1^ corresponds to the –COO group, and the peaks at 1107.29 and 548.55 cm^−1^ correspond to the –CO and Se-O groups [44,45]. The obtained FTIR spectra were similarly detected in other selenium particles [26]. The surface charge of the SeNPs was analyzed by zeta potential, confirming that SeNPs are negatively charged, as shown in Figure 1d. The surface charge of the SeNPs was −13.9 mV, which is mainly due to the presence of –COO, –CO, and –OH chemical groups on the surface, as confirmed by FTIR assessment. It is worthy to note that higher zeta potential was suitable for stable particle colloidal suspensions. Finally, the optical properties of the SeNPs were analyzed by UV-visible spectroscopy. The absorption spectra of the SeNPs are presented in Figure 1e. The SeNPs exhibited a broad absorption peak at 325.24 nm.

### 3.2. TMB-Based Oxidase-like Activity

Antioxidant effect without any supplement is a unique property of selenium-contained particles [26,46,47]. Thus, for investigating antioxidant effect of SeNPs, TMB-based oxidase-like activity was performed. Figure 1f shows optical images of TMB and SeNPs, with a concentration of 200 µg/mL for both and a typical blue color after 1 h and 5 h of reaction was noticed. The change in color of SeNPs in TMB solution to blue in the presence of H_2_O_2_ confirms the oxidase-like activity of the SeNPs. Initially (i.e., t = 0), no color was observed, while over time, the blue color (t = 2 h) became deep blue (t = 5), indicating the time-dependent oxidase-like activity of SeNPs. Quantitative analysis of the oxidase-like activity was measured by UV-visible spectroscopy and an absorbance peak was observed at 652 nm. This peak appeared due to the oxidization of TMB. Figure 1f shows the UV-visible spectra for TMB (t = 0) and SeNPs with varying time (t = 0 h, 1 h, and 5 h). The UV-visible absorption value (i.e., the intensity of peak) increased with the reaction time, confirming the intrinsic oxidase-like activity of SeNPs. Previously, we showed that the oxidase-like activity of SeNPs increases with increasing nanoparticle concentration at a constant time [26]. Furthermore, an oxidase-like activity commonly increases with reaction time and nanoparticle dose [48]. Overall, our results suggest that 200 µg/mL of SeNPs can achieve effective oxidase activity in 5 h of reaction.

### 3.3. Effect of Selenium Nanoparticles on Cell Viability in C2C12-Induced ROS Conditions

**Figure 2:**
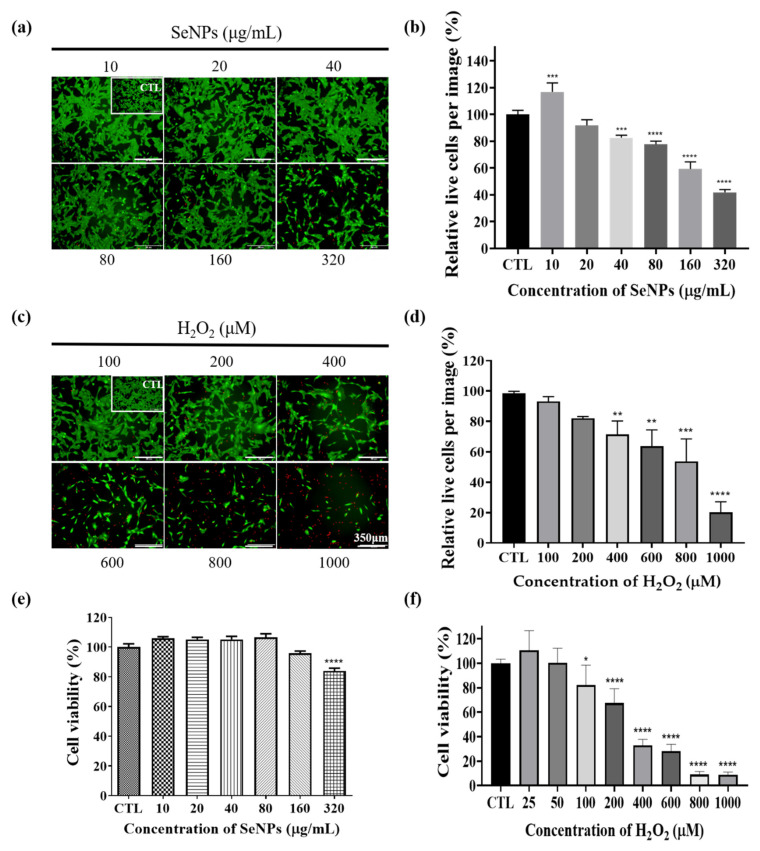
Cell viability was determined by live (green) and dead (red)-staining, and CCK-8 assays. After cell seeding for 24 h, C2C12 cells were analyzed using CCK-8 solution and stained using live/dead solution to evaluate viability. (**a**) Live/dead-staining following treatment with various SeNPs concentrations. (**b**) The quantitative live/dead assay results, which were treated with SeNPs. (**c**) Live/dead-staining following treatment with various H_2_O_2_ concentrations. (**d**) The quantitative live/dead assay results, which were treated with H_2_O_2_. (**e**) The relative cell viability of C2C12 cells cultured in different SeNPs concentrations. (**f**) Viability of C2C12 cells treated with various concentrations of H_2_O_2_. All data was normalized by control cell numbers. The statistical significance of (**e**,**f**) was calculated using one-way analysis of variance (ANOVA), followed by a two-sided Dunnett’s multiple comparison test compared to the control (CTL; scale bar = 350 μm). * Represents *p* < 0.05, ** *p* < 0.01, *** *p* < 0.001, and **** *p* < 0.0001; *n* = 3., * compared with the SeNPs and H_2_O_2_-untreated groups.

**Figure 3:**
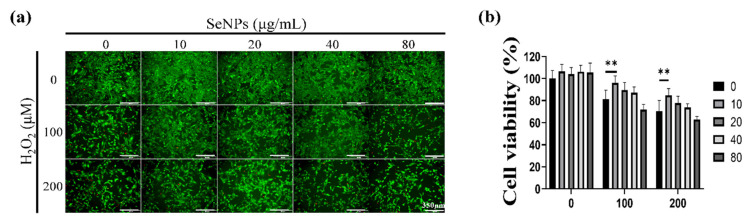
Cell viability was determined by live (green) and dead (red)-staining, and CCK-8 assays. After cell seeding for 24 h, C2C12 cells were analyzed using CCK-8 solution and stained using live/dead solution to evaluate viability. (**a**) Live/dead-staining following treatment with various SeNPs concentrations and H_2_O_2_. (**b**) Viability of C2C12 cells treated with various concentrations of SeNPs and H_2_O_2_. The statistical significance of (**b**) was calculated using two-way ANOVA, followed by a two-sided Dunnett’s multiple comparison test compared to untreated groups (scale bar = 350 μm). ** Represents *p* < 0.01.

Thirty-two milligrams of SeNPs were quantified and dissolved in PBS. Then, the selenium stock was made to the highest concentration of 320 μg/mL and used to treat C2C12 cells, followed by 2-fold serial dilutions. As a result of treatment with SeNPs, non-toxic C2C12 cell viability over 100% was observed at a concentration of 80 μg/mL or less (Figure 2a,b,e). All data was normalized by control cell numbers. Thus, up to 80 μg/mL was chosen for the next in vitro experiment. In the case of C2C12 cells treated with hydrogen peroxide to mimic the high-ROS condition, cells survived less in all H_2_O_2_ concentrations from 100 μM (Figure 2c,d,f). To observe the SeNPs’ cell salvage effect as antioxidant, SeNPs and 100–200 μM of H_2_O_2_ were co-treated (Figure 3). SeNPs could increase cell viability at 10 μg/mL from both 100 and 200 μM of H_2_O_2_ challenge conditions, eliciting the protective role against muscle progenitor cells along with other cell types [26,49]. In addition, 20 μg/mL of SeNPs revealed a high average value in cell viability compared to the non-SeNPs-treated condition without statistic difference under H_2_O_2_ challenge conditions, while higher concentrations showed similar cell viability. Since long-term SeNPs treatment during differentiation might affect cell viability, the highest concentration was set to 20 μg/mL for further studies, including the differentiation study, and this amount was similarly used in other literature [50].

### 3.4. ROS-Staining

**Figure 4:**
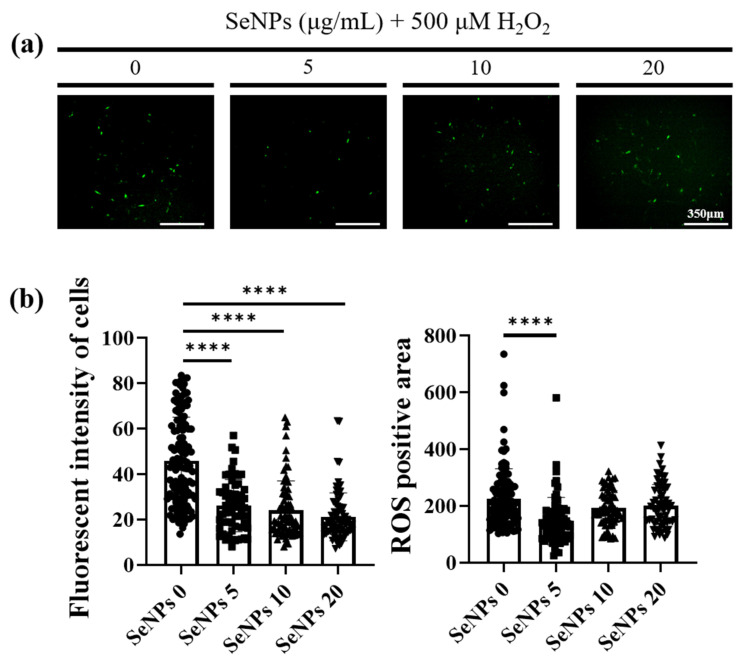
C2C12 cells were exposed to 500 μM of H_2_O_2_ to induce oxidative stress and then were recovered by culturing in a medium with or without SeNPs. High oxidative stress conditions were enabled by pretreatment with H_2_O_2_ for 4 h. (**a**) SeNP treatment reduced the levels of reactive oxygen species (ROS). (**b**) The fluorescence intensity of cells and ROS-positive areas were measured using ImageJ. Statistical significance was calculated using one-way ANOVA, followed by a two-sided Dunnett post hoc test compared to the untreated SeNPs group (scale bar = 350 μm). **** Represents *p* < 0.0001; *n* = 5.

As high levels of ROS have been reported to induce apoptosis and inhibit the differentiation of muscle progenitor cells during repair and regeneration [51], ROS-staining was analyzed to confirm the ability of SeNPs to regulate ROS. To measure intracellular ROS production, we used 5-(and-6)-carboxy-29,79-dichlorodihydrofluorescein diacetate (carboxy H2DCFDA) after SeNPs and 500 μM of H_2_O_2_ treatment. In total, 500 μM of H_2_O_2_ was selected to maximize the ROS production within the limitation of ROS dye sensitivity and to investigate the antioxidant role of SeNPs in a high-ROS environment [26,52]. The ROS production was confirmed at the 4th hour because it showed toxicity after 24 h. In addition, we wanted to confirm the dramatic cytoprotective and antioxidant effects at high H_2_O_2_ concentrations, such as 500 μM, and the low intensity of ROS-staining at 200 μM, confirming cell viability. As shown in Figure 4a, control cells showed a large number of fluorescent cells. In contrast, the cells treated with 5 μg/mL of SeNPs showed weak fluorescence, indicating that 5 μg/mL of SeNPs efficiently controlled ROS. Furthermore, it has been suggested that ROS may affect several cellular activities. Figure 4b shows the results obtained by analyzing the intensity and positive areas of staining using ImageJ. As a result of the intensity analysis of fluorescently stained cells, the SeNP-treated group showed a lower intensity than the untreated group and the highest decrease was observed at 5 μg/mL of SeNPs. ROS generated through various extracellular and intracellular actions attract attention as novel signaling mediators involved in the growth, differentiation, progression, and death of cells [53]. Previous studies have shown that another type of stress (heat) possibly causes overproduction and accumulation of ROS, resulting in damage to cells, and SeNPs could protect the apoptosis of C2C12 induced by high ROS [49]. These results, in addition to previous studies revealing the therapeutic role of SeNPs as antioxidants, suggest that SeNPs protect cells by regulating ROS and may be involved in various cellular activities [54,55].

### 3.5. Effect of Selenium Nanoparticles on the Expression of Myogenic Genes Determined by qRT–PCR

**Figure 5:**
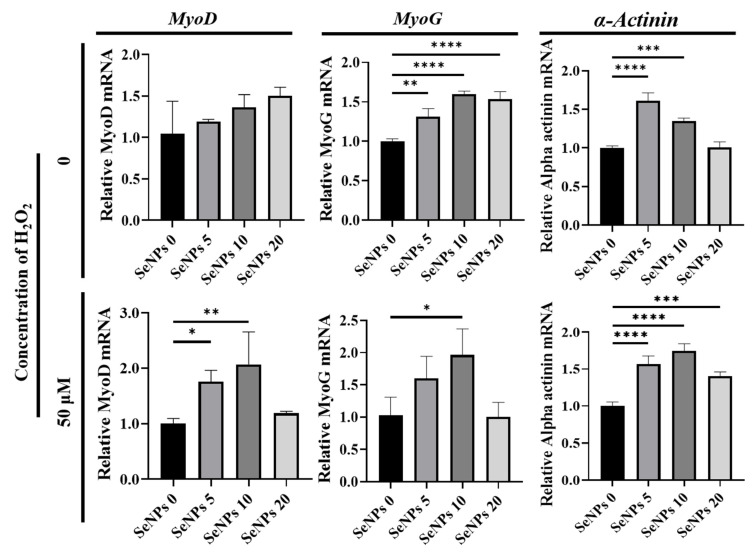
The effect of SeNPs on the expression of myogenic genes through qRT–PCR analysis. The relative expression levels of target genes normalized to *Gapdh* were calculated using the delta cycle threshold (Ct) method. The figure shows the relative expression of multiple genes relative to gene expression in the negative control treatment cells. The results of the qRT-PCR analysis of myogenic markers five days after treatment with myogenic differentiation media. In the group treated with selenium, the activities of *MyoD*, *MyoG*, and *α-actinin* were higher than those in the group not treated with selenium. Statistical significance was calculated using one-way ANOVA, followed by a two-sided Dunnett post hoc test compared to the untreated SeNPs group * Represents *p* < 0.05, ** *p* < 0.01, *** *p* < 0.001, and **** *p* < 0.0001; *n* = 3.

To confirm the effect of SeNPs, qRT-PCR was used to investigate the myogenic-relative mRNA expression levels such as *MyoD*, one of the earliest markers of myogenesis; *MyoG*, known as a transcription factor for myoblast differentiation; and *α-Actinin*, which is involved in the assembly and maintenance of muscle fibers. Figure 5 shows that SeNPs effectively up-regulated myogenic-relative genes after 5 days of culture in differentiation media with or without H_2_O_2_. In the absence of H_2_O_2_, the expression of the *MyoD* gene did not show any significant differences. The expression of the *MyoG* gene was 1.32 ± 0.1–1.60 ± 0.04 in all SeNP-treated groups and *α-Actinin* gene expression increased to 1.61 ± 0.1 and 1.35 ± 0.04 when the cells were treated with 5–10 μg/mL of SeNPs. Under H_2_O_2_ conditions, the expression of the *MyoD* gene increased to 1.77 ± 0.29 and 2.39 ± 0.25 when treated with 5–10 μg/mL of SeNPs. The expression of the *MyoG* gene was 1.73 ± 0.06 in the group treated with 10 μg/mL of SeNPs and the *α-Actinin* gene increased to 1.39 ± 0.08~1.78 ± 0.1 in all groups treated with SeNPs. In addition to the previous study which found that antioxidant ascorbic acid (200 μM) can promote the differentiation of C2C12 cells, it was confirmed that SeNPs (5–20 μg/mL) could also be effective in differentiation promotion in C2C12 cells [56]. Along with the therapeutic result of seleno-proteins to myogenic cells challenged with heat stress-induced high ROS, in which *MyoD* and *MyoG* were upregulated in the presence of selenoproteins [57], it was confirmed that not only heat stress but also ROS could be modulated to influence differentiation.

### 3.6. Effect of Selenium Nanoparticles on Myotube Formation

**Figure 6:**
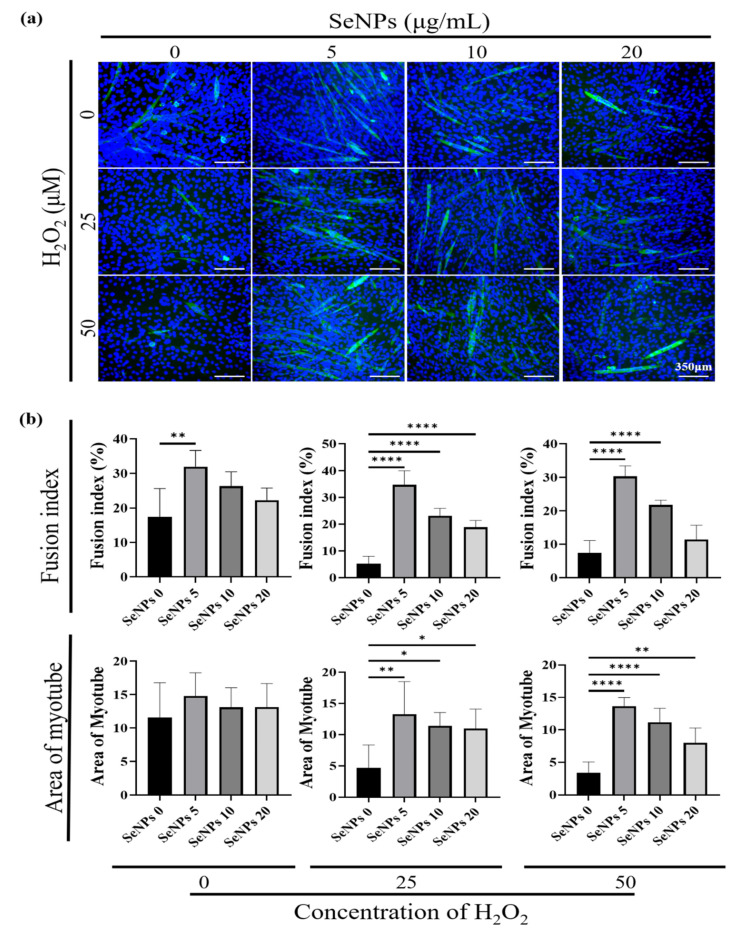
Myogenic differentiation of C2C12 cells according to SeNPs treatment. (**a**) Image using MHC after 5 days of differentiation induced by SeNPs treatment. (**b**) Quantification of myogenic differentiation. Analysis was performed using ImageJ software. Statistical significance was calculated using one-way ANOVA, followed by a two-sided Dunnett post hoc test compared to the untreated SeNPs group * Represents *p* < 0.05, ** *p* < 0.01, and **** *p* < 0.0001; *n* = 3.

To evaluate whether SeNPs are effective in the differentiation of C2C12 cells, immunocytochemistry (ICC)-staining was performed on the 5th day after replacement with myogenic differentiation media (Figure 6a). To quantify the degree of differentiation, the myotube fusion index was calculated by the proportion of nuclei in myotubes to the total nuclei. To quantify and analyze the degree of differentiation of C2C12 cells, the fusion index and area of the myotube were analyzed. First, in the H_2_O_2_ untreated group, the fusion index increased by approximately 14% in the 5 μg/mL of the SeNPs-treated group compared to the untreated SeNPs group and no significant differences were found in the area. In the 25 μM of the H_2_O_2_ treatment group, the fusion index increased by approximately 13–29% in the SeNPs treatment group and the area increased by approximately 6–9. In the 50 μM of the H_2_O_2_ treatment group, the fusion index increased by approximately 14–23% in the 5–10 μg/mL of the SeNPs treatment group and the area increased by approximately 4–10 (Figure 6b). From these results, we confirmed that the expression of myogenic markers increased upon SeNPs treatment compared to the untreated group and also SeNPs can be involved in myogenesis by regulating ROS even in a ROS environment induced by H_2_O_2_. In a previous study, heat stress induced the downregulation of the C2C12 gene and the seleno-protein had a potential protective effect under heat stress [49]. In addition, we confirmed the potential protective effect against damage to C2C12 cells and the upregulation of both the gene and protein expression even in the ROS environment.

### 3.7. Western Blot

**Figure 7:**
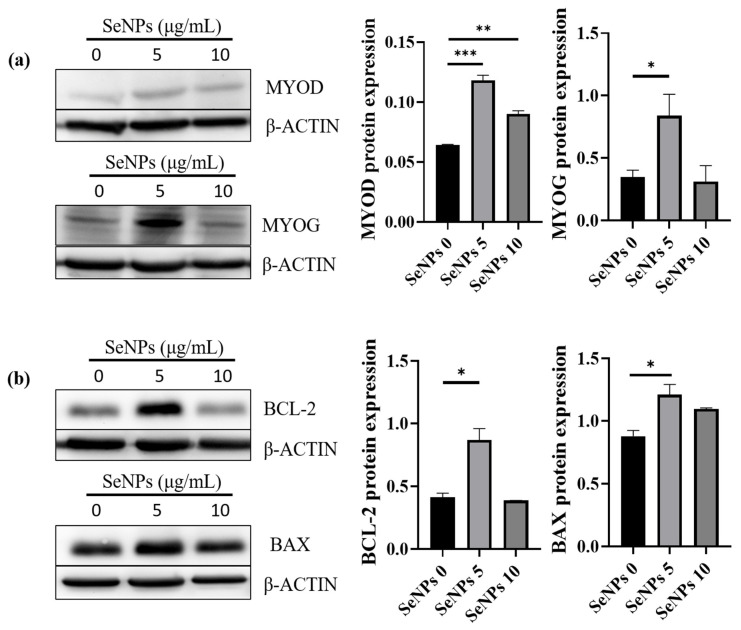
SeNPs upregulate myogenic markers during C2C12 cell differentiation and prevent apoptosis. (**a**) SeNPs promote myogenic differentiation and (**b**) increase cell viability by protecting C2C12 cells under 50 μM of H_2_O_2_ for 5 days. Statistical significance was calculated using one-way ANOVA, followed by a two-sided Dunnett post hoc test compared to the untreated SeNPs group. * Represents *p* < 0.05, ** *p* < 0.01, and *** *p* < 0.001; *n* = 3.

It was confirmed that SeNPs protect cells from ROS and promote differentiation in the ROS condition. Next, we identified major apoptotic signaling molecules, such as BAX and BCL-2, and major myogenesis signaling molecules, such as MYOD and MYOG, by western blot analysis. This experiment was conducted at a concentration of 5–10 μg/mL of SeNPs, which confirmed a higher level of differentiation through ICC-staining with MHC. In the case of MYOD, the SeNP-treated group showed a significant increase in expression level compared to the untreated group (Figure 7a). However, in the case of MYOG, only the 5 μg/mL of the SeNPs treatment group showed an increase in expression (Figure 7a). Similar to the myogenic effect from another natural antioxidant (curcumin, 1 μM) displaying enhanced MHC (muscle differentiation marker), we observed enhancement of myogenic proteins [58]. In the case of BCL-2, an antiapoptotic factor, a significant increase was confirmed in the 5 μg/mL of the SeNPs treatment group (Figure 7b); in the case of BAX, the 5 μg/mL of the SeNPs treatment group showed similar results to the untreated group (Figure 7b). These data show not only enhanced expression of markers at the RNA level but also enhanced expression at the protein level. In support of previous studies showing that SeNPs play an important role in ROS regulation by analyzing apoptosis signals [32,35], their role in protecting cells from ROS and promoting myoblast differentiation was confirmed.

## 4. Conclusions

**Figure 8:**
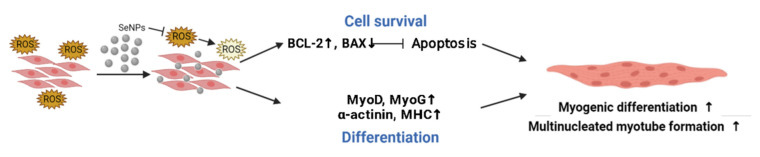
Schematic diagram of C2C12 cells cultured and treated with SeNPs for myogenic differentiation. SeNPs treatment protects cells from ROS (orange is higher ROS; yellow is lower ROS), thereby increasing cell viability and promoting cell differentiation, contributing to multinucleated myotube formation.

The purpose of this study was to investigate the efficiency of apoptosis protection and differentiation promotion through ROS regulation of SeNPs in the ROS environment in C2C12 cells. The findings of this study are schematically presented in Figure 8, which illustrates a series of antioxidant effects of the SeNPs during the myogenic differentiation. This study revealed that SeNPs effectively restored C2C12 cells from the constant H_2_O_2_ exposure environment via down-regulation of ROS and enhanced myogenic differentiation in both normal and high-ROS conditions. In particular, SeNPs increased myogenic-related mRNA levels (*MyoD*, *MyoG*, and *α-actinin*) and promoted multinucleated mature myoblasts. Additionally, SeNPs enhanced the production of the myogenic protein (MYOD and MYOG) and anti-apoptotic protein (BCL-2). Collectively, it can be concluded that SeNPs showed the potential for skeletal muscle repair by acceleration of myogenic differentiation via tuning the ROS production. Along with the ROS regulatory pathway of SeNPs in the myogenic differentiation condition, the clinical effectiveness of SeNPs for muscle tissue regeneration needs to be reinforced in future animal studies.

## Data Availability

The data presented in this study are available in article.
